# Indwelling catheters increase altered mental status and urinary tract infection risk: A retrospective Cohort Study

**DOI:** 10.1016/j.amsu.2021.102186

**Published:** 2021-03-06

**Authors:** Toko Fukushima, Kazuhiro Shoji, Atsuko Tanaka, Yukari Aoyagi, Shoko Okui, Marie Sekiguchi, Ayako Shiba, Takanori Hiroe, Yasushi Mio

**Affiliations:** a Tokyo Jikei University Katsushika Medical Center, Department of Anesthesiology, Tokyo, Japan; b The Jikei University School of Medicine, Department of Anesthesiology, Tokyo, Japan; c Kyoto University Graduate School of Medicine, Department of Biostatistics, Kyoto, Japan

**Keywords:** Urinary catheter, Perioperative complication, Delirium, Urinary tract infection, AKI, acute kidney injury, ASA-PS, American Society of Anesthesiology physical status, ASD, absolute standardized differences, CAM-ICU, Confusion Assessment Method for the ICU, CDC, Center for Disease Control and Prevention, CI, confidence interval, ICU, intensive care unit, IPW, inverse probability weighting, IUCs, indwelling urinary catheters, OR, odds ratio, SCr, serum creatinine levels

## Abstract

**Background:**

Although indwelling urinary catheters (IUCs) are used intraoperatively and may cause complications (*e.g.*, delirium), only few robust studies have investigated the association between intraoperative IUC use and complications. We hypothesized that IUC use might increase the postoperative incidence of altered mental status and/or urinary catheter infection.

**Materials and methods:**

In this retrospective single-center cohort study, we analyzed the data of adult patients undergoing surgery at our facility between January 2013 and December 2018. The primary endpoint was altered mental status and/or incidence of urinary catheter infections. The patients were divided into IUC and control groups. A multivariable logistic regression model was used to identify the predictors of postoperative complications, and a multivariable Cox proportional hazards regression model was used to analyze hospital discharge in unmatched and inverse propensity-weighted patients.

**Results:**

Of the 14,284 patients that were reviewed, we analyzed 5112 patients (control group, 44.0%; IUC group, 56.0%). Almost all procedures comprised less invasive surgeries. The prevalence of postoperative altered mental status and postoperative urinary catheter infection were 3.56% and 0.04%, respectively. After inverse propensity weighting, all baseline characteristics were similar between the two groups. However, patients with IUCs had a higher risk of postoperative complications (adjusted odds ratio, 1.97; 95% confidence interval [CI], 1.50–2.59) and prolonged hospital stays (hazard ratio, 0.84; 95% CI, 0.80–0.89).

**Conclusion:**

In patients undergoing less invasive surgery, IUCs may be associated with a relatively high risk of altered mental status or urinary catheter infection. These data may facilitate preoperative discussions regarding the perioperative use of IUCs.

## Highlights

• IUCs may increase the incidence of altered mental status postoperatively.
• IUCs may increase the incidence of urinary catheter infections postoperatively.
• IUC use might be a risk factor for prolonged hospital stay.
• Our results may facilitate preoperative discussions regarding perioperative IUC use.

## 1 Introduction

During surgery, indwelling urinary catheters (IUCs) are frequently used to assess urine output as an indicator for the optimal blood pressure [[1], [2], [3], [4], [5], [6], [7]] and a predictor of postoperative acute kidney injury (AKI) [[8], [9], [10]]. Worldwide, nearly 80% of all anesthesiologists measure the urine output [8,[11], [12], [13], [14], [15], [16], [17]]. However, IUCs can cause complications, most frequently catheter-induced urinary tract infections. Approximately 12–16% of adult hospital inpatients will require an IUC, and a patient has a 3–7% increased risk of acquiring a catheter-associated urinary tract infection each day the IUC remains [18]. The Center for Disease Control and Prevention (CDC) considers an IUC indication appropriate when one or more of the following criteria are met: a patient undergoing urologic or other surgery on contiguous structures of the genitourinary tract; anticipated prolonged surgery duration, catheters inserted for this reason should be removed in the post-anesthesia care unit; anticipated need for large-volume infusions or diuretics during surgery; or need for intraoperative measuring of the urinary output [18]. However, these suggestions were not based on robust evidence evaluating risks (*e.g.*, delirium, urinary tract infection) and benefits (*e.g.*, maintaining optimal blood pressure, decreased incidence of postoperative AKI) of intraoperative IUC use.

Some studies reported that perioperative IUC usage may induce discomfort, delirium, or urinary tract infection [[19], [20], [21], [22], [23], [24]]. Almost all studies examining perioperative IUC usage had low evidence levels because delirium and urinary tract infection were not primary endpoints. Eide et al. [22] demonstrated that urinary catheter use is associated with delirium. However, their study population comprised only patients undergoing transcatheter aortic valve implantation [22]. Therefore, the benefit-risk assessment of intraoperative IUC usage is inconclusive.

Kuriyama et al. [25] reported that only 5.3% of the elective surgical patients from seven participating intensive care units (ICUs) in Japan had an appropriate IUC based on indications suggested by the CDC. Eide et al. [22] also suggested that studies should evaluate whether IUC use could be avoided entirely in patients with transcatheter aortic valve implantation. Therefore, we hypothesized that perioperative IUC use might increase the incidence of altered mental status and/or urinary tract infection postoperatively but might not decrease that of postoperative AKI. Before we conduct a randomized controlled trial (RCT) to test this hypothesis, we supposed that we should estimate the incidence of altered mental status or urinary tract infection postoperatively associated with IUCs. For this reason, we conducted the current retrospective cohort study that may be helpful to determine whether intraoperative IUC use is predominantly a risk or a benefit.

## 2 Methods

The Institutional Review Board of our institution approved the study and waived the requirement for informed consent because the procedures comprised no additional interventions and because all patient data were anonymous. This retrospective cohort study was registered with UMIN-ICDR (UMIN000042664, https://upload.umin.ac.jp/cgi-bin/ctr/ctr_view_reg.cgi?recptno=R000048679) in accordance with the declaration of Helsinki. The opt-out method was available on the hospital's website. This study was conducted following the Strengthening the Reporting of Cohort Studies in Surgery (STROCSS) [26] and the Strengthening the Reporting of Observational Studies in Epidemiology (STROBE) guidelines. We included all adult (age ≥18 y) noncardiac surgical patients who required general anesthesia between January 1, 2013, and December 31, 2018. We excluded the following patients: patients undergoing urology surgery because IUCs were needed during and after surgery (to assess complications, urine output, and to prevent complications), patients who needed an IUC during surgery (to assess the urine output, the indicators for optimal blood pressure were as follows: emergency surgery, mechanical ventilation before surgery, and carotid endarterectomy; for assessing urine drainage because of urination disorder, the indicators were as follows: traumatic spinal cord injury, epidural catheter use, spinal anesthesia, severe motor and intellectual disabilities), patients undergoing neurosurgery affecting postoperative consciousness and immobility (craniotomy, trepanation, intracranial endovascular therapy), patients who could not speak and who could not vocalize unfamiliar words (tracheostomy), patients undergoing chronic hemodialysis due to reduced urine output, and patients who experienced cardiac arrest before surgery.

Patient characteristics and intraoperative data were retrieved from the operating room database (ORSYS, Philips Electronics Japan, Tokyo, Japan) and the patient information system (PIMS, Philips Electronics Japan, Tokyo, Japan; HOPE EGMAIN-GX, FUJITSU, Kanagawa, Japan) of the hospital. We did not collect post-hospital discharge data because those were not recorded routinely.

The primary endpoint was the incidence of altered mental status and/or urinary tract infection. The secondary endpoints were the length of postoperative hospital stay and AKI incidence after surgery.

We use the expression “altered mental status” instead of delirium because we usually do not employ delirium diagnostic tools such as the Confusion Assessment Method for the ICU (CAM-ICU); thus, this information was not available. Before and after conducting this study, the descriptions of altered mental status in Japanese were discussed by seven anesthesiologists to decide whether these descriptions reflected altered mental status and not an arbitrary selection. Nursing records between the time of discharge from the operating room and the next day at 23:59 were analyzed.

The definition of a urinary tract infection was based on the CDC guidelines [27]. A patient had to meet both of the following criteria: presence of at least one of the following signs or symptoms: fever **(** > **38.0°C)**, suprapubic tenderness, costovertebral angle pain or tenderness, urinary urgency, urinary frequency, and dysuria and identification of no more than two species of organisms from urine culture, at least one of which was a bacterium with a load of ≥10^5^ colony-forming units/mL. Postoperative AKI was defined according to the Kidney Disease Improving Global Outcomes guidelines [8]. Severity levels were defined based on changes in serum creatinine levels: stage 1, increase in serum **creatinine levels (sCr)** of ≥0.3 mg/dL within 48 h or an increase corresponding to 1.5–1.9-times the baseline value within 7 d; stage 2, increase in **sCr** corresponding to 2.0–2.9-times the baseline value; and stage 3, increase in **sCr** of ≥4.0 mg/dL, an increase corresponding to >3.0-times the baseline value, or the initiation of or increase in the dose of renal replacement therapy within 7 d. Urine output criteria were not used in the present study.

### 2.1 Sample size

Our sample size estimate was based on the expected incidence of postoperative altered mental status or urinary tract infection as suggested in previous studies [18,20,24,[28], [29], [30], [31]]. Assuming a baseline incidence of the primary endpoints of around 20% in the control group and considering a 15% increase in incidence in the IUC group to be clinically important, we estimated that a total of 1892 patients would be required to demonstrate this difference **(type 1 error** < **0.05 and a power of 0.8)**. We expected approximately 5000 eligible patients to visit the medical center over a 6-year period.

### 2.2 Adjustment for differences between groups

All patients were divided into two groups (IUC group and control group) according to intraoperative IUC use. In our institution, the need for IUC during surgery was decided by the physician or anesthesiologist. The urinary catheter was inserted by either a trained physician or by the nurses. We supposed that characteristics would differ between the two groups. Therefore, inverse probability weighting based on the propensity-score was used as the primary tool to adjust for differences [32]. We constructed a logistic regression model to calculate the propensity-score for each subject who underwent intraoperative IUC placement based on risk factors for cardiovascular disease and postoperative AKI [33]. Preoperative variables that would affect the indication for IUC use were as follows: age; sex; body mass index (kg/m^2^); American Society of Anesthesiology physical status (ASA-PS) score; surgical specialty; the serum creatinine level (mg/dL) before surgery; a history of dual antiplatelet therapy, defined as the use of aspirin or cilostazol combined with a P2Y12 inhibitor; a history of using oral anticoagulant medications, defined as warfarin or direct oral anticoagulants; and mean blood pressure on admission to the operating room (mmHg), a non-invasive blood pressure measurement excluding pressure pulse values < 20 mmHg, which were considered measuring errors. Covariate balances before and after inverse probability weighting were compared using standardized differences [33]. Absolute standardized differences **(ASD)** within 10% for all variables were indicative of successful balancing.

### 2.3 Missing data imputation

We filled in missing data with multiple imputations using the R package “mice version 3.6.0” (R Core Team, 2019) [27]. To predict missing values, we used information about intervention allocation (with or without IUC use) and the variables independently associated with postoperative hospital stay in the multiple logistic regression analysis.

### 2.4 Statistical analysis

Baseline characteristics for the full cohort are summarized as numbers and percentages for categorical variables and as median values and interquartile ranges for continuous variables. Postoperative variables before and after inverse probability weighting **(IPW)** were analyzed for between-group differences using the Wilcoxon-Mann-Whitney test for continuous variables, Fisher's exact test for categorical variables, and the log-rank test for postoperative hospital stay. A two-tailed P value < 0.05 was considered statistically significant. A multivariable logistic regression model was used to identify the predictors of the primary endpoints, and a multivariable Cox proportional hazards regression model was used for analyzing postoperative hospital stay. We used Akaike's Information Criteria for goodness-of-fit measures. Kaplan–Meier curves were used to show the incidence of hospital discharge from the day of surgery through 30 d.

Several sensitivity analyses were performed. First, patient matching based on the propensity-score was performed to adjust for differences. Second, we constructed a doubly robust strategy combining IPW with a logistic regression model [34]. All statistical analyses were performed using R (version 3.6.1, R Foundation for Statistical Computing, Vienna, Austria).

## 3 Results

**Fig. 1:**
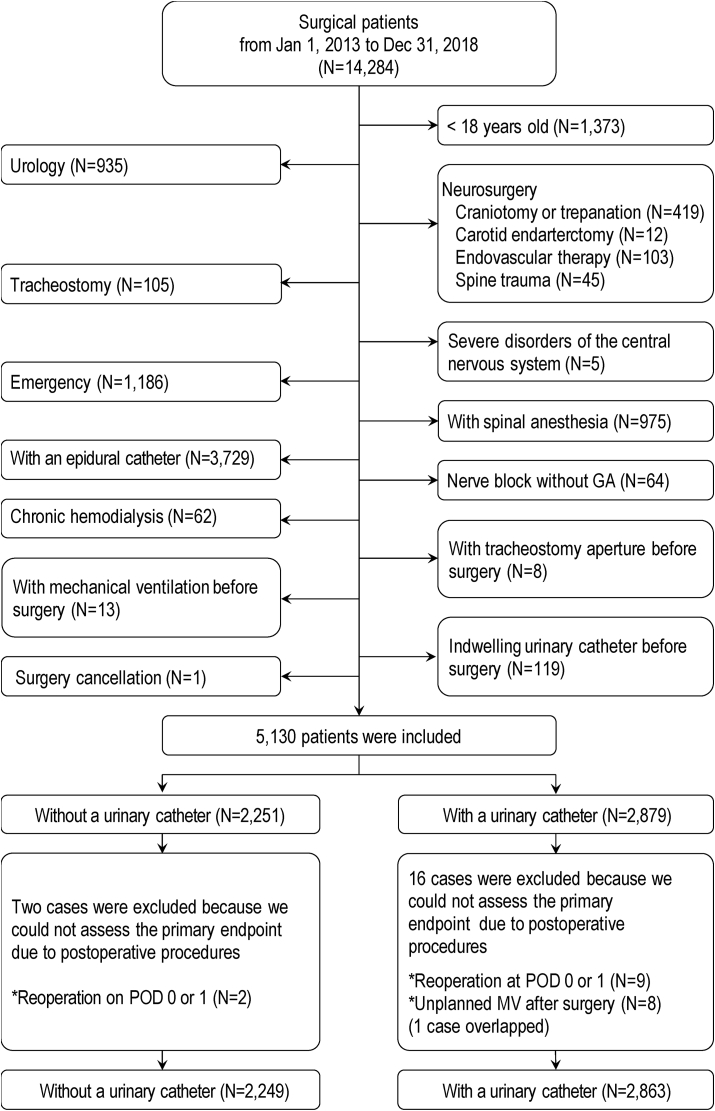
Patient enrollment procedure and study flow chart. Abbreviations: GA, general anesthesia; MV, mechanical ventilation; POD, postoperative day.

Between January 1, 2013, and December 31, 2018, 14,284 surgical patients were managed by an anesthesiologist. Of those, 5130 patients met the inclusion criteria. We excluded 18 patients because we could not assess the primary endpoint and we think that the procedures were not associated with IUC use (10 patients received reoperation, and 9 patients received mechanical ventilation within 24 h after surgery with 1 patient overlap; all 18 patients had an IUC). Patients who had re-surgery would have received an IUC because re-surgery was an emergency and we usually maintain urine output as a sign of optimal blood pressure control in these cases. Patients who had mechanical ventilation would have been usually restricted, and IUCs tend to be used for immobilization. Thus, we analyzed data from 5112 patients (Fig. 1). We considered the type of missing data to be missing at random. Seven variables (height, weight, body mass index, mean blood pressure before surgery, serum creatinine before surgery, postoperative AKI, and duration of surgery and bleeding) with 1526 missing data were finally imputed.

The pre- and intraoperative characteristics of the unmatched patients are summarized in Table 1, Table 2. The patients of the IUC group were older than those of the control group **(ASD = 0.477)**. The predicted **(ASD = 0.162)** and actual **(ASD = 0.912)** durations of surgery were longer in the IUC group compared to those in the control group. IUCs were used more often in general surgery **(ASD = 0.498).** The percentage of patients with an ASA-PS score of 1 was higher in the control group than in the IUC group **(ASD = 0.363)**.

**Table 1:** Baseline demographics of all patients and inverse propensity-weighted patients.

	All patients				Inverse propensity-weighted patients			
	Control	IUC	ASD	*P*-value	Control	IUC	ASD	*P*-value
Number of patients, N (%)	2249	2863			3080.94	2863.00		
Age, y	48 [36–63]	60 [44–72]	0.477	<0.001	60 [47–71]	60 [44–72]	0.024	0.808
Male, N (%)	1090 (48.5)	997 (34.8)	0.279	<0.001	1229.9 (39.9)	997.0 (34.8)	0.105	0.23
Body mass index, kg·m^−2^	22.8 [20.6–25.5]	23.1 [20.6–25.9]	0.073	0.034	23.0 [20.5–25.9]	23.1 [20.6–25.9]	0.021	0.916
ASA physical status score			0.363	<0.001			0.107	0.283
1, N (%)	1101 (49.0)	928 (32.4)			868.4 (28.2)	928.0 (32.4)		
2, N (%)	1067 (47.4)	1709 (59.7)			1999.0 (64.9)	1709.0 (59.7)		
3, N (%)	81 (3.6)	226 (7.9)			213.5 (6.9)	226.0 (7.9)		
Surgery type, N (%)			0.498	<0.001			0.089	0.571
General surgery^a^, N (%)	633 (28.1)	1283 (44.8)			1393.3 (45.2)	1283.0 (44.8)		
ENT surgery^b^, N (%)	820 (36.5)	498 (17.4)			457.3 (14.8)	498.0 (17.4)		
Orthopedic surgery^c^, N (%)	724 (32.2)	896 (31.3)			979.3 (31.8)	896.0 (31.3)		
Others, N (%)	72 (3.2)	186 (6.5)			251.1 (8.1)	186.0 (6.5)		
Preoperative MBP, mmHg	92 [83–101]	92 [83–102]	0.055	0.05	92 [83–101]	92 [83–102]	<0.001	0.811
Creatinine, mg/dL	0.71 [0.6–0.8]	0.68 [0.6–0.8]	0.019	0.52	0.70 [0.6–0.81]	0.68 [0.59–0.81]	<0.001	0.127
Predicted duration of surgery, min	150 [120–180]	180 [150–240]	0.857	<0.001	180 [150–240]	180 [150–240]	0.069	0.732

Notes: Data are presented as number (percentage) or median [interquartile range].

**Abbreviations:** ASD, absolute standardized difference; ASA, American Society of Anesthesiology; ENT, ear, nose, and throat; IUC, indwelling urinary catheter; MBP, mean blood pressure.

a General surgery mainly consisted of cholecystectomy, hernia repair, and mastectomy.

b ENT surgery mainly consisted of septoplasty, tonsillectomy, tympanoplasty, and parotidectomy.

c Orthopedic surgery mainly consisted of treatment for upper or lower limb fracture, arthroplasty or osteotomy of the knee or foot, and spine surgery.

**Table 2:** Intraoperative characteristics and outcomes of all patients and inverse propensity-weighted patients.

	All patients				Inverse propensity-weighted patients			
	Control	IUC	ASD	*P*-value	Control	IUC	ASD	*P*-value
Anesthesia			0.061	0.103			0.105	0.057
Inhalation, N (%)	1956 (87.0)	2441 (85.3)			2726.0 (88.5)	2441.0 (85.3)		
TIVA, N (%)	274 (12.2)	384 (13.4)			334.4 (10.9)	384.0 (13.4)		
Others, N (%)	19 (0.8)	38 (1.3)			20.5 (0.7)	38.0 (1.3)		
Nerve block, N (%)	1082 (48.1)	1767 (61.7)	0.276	<0.001	1668.2 (54.1)	1767.0 (61.7)	0.154	0.052
Use of fentanyl, N (%)	2122 (94.4)	2772 (96.8)	0.12	<0.001	2960.5 (96.1)	2772.0 (96.8)	0.04	0.325
Use of remifentanil, N (%)	1264 (56.2)	1979 (69.1)	0.27	<0.001	2015.1 (65.4)	1979.0 (69.1)	0.079	0.213
Duration of surgery, min	83 [55–113]	131 [95–178]	0.912	<0.001	109 [80–161]	131 [95–178]	0.344	<0.001
Urine output, mL	150 [80–300]	153 [70–310]	0.063	0.615	150 [110–250]	153 [70–310]	0.11	0.595
UC removal in PACU, N (%)	–	37 (1.3)	0.162	<0.001	–	37 (1.3)	0.162	<0.001
Primary endpoint								
AMS or UTI	41 (1.8)	143 (5.0)	0.175	<0.001	98.3 (3.2)	143.0 (5.0)	0.091	0.397
AMS, N (%)	41 (1.8)	141 (4.9)	0.172	<0.001	98.3 (3.2)	141.0 (4.9)	0.088	0.412
PACU, N (%)	20 (0.9)	77 (2.7)	0.136	<0.001	23.5 (0.8)	77.0 (2.7)	0.148	<0.001
General ward, N (%)	24 (1.1)	94 (3.3)	0.152	<0.001	77.4 (2.5)	94.0 (3.3)	0.046	0.691
UTI, N (%)	0 (0)	2 (0.1)	0.037	0.588	0 (0.0)	2.0 (0.1)	0.037	0.143
Secondary endpoint								
KDIGO AKI, N (%)	7 (0.3)	7 (0.2)	0.013	0.854	7.6 (0.2)	7.0 (0.2)	<0.001	0.996
Stage 1, N (%)	7 (0.3)	6 (0.2)			7.6 (0.2)	6.0 (0.2)		
Stage 2, N (%)	0 (0)	0 (0)			0 (0)	0 (0)		
Stage 3, N (%)	0 (0)	1 (0)			0 (0)	1.0 (0)		
Postoperative stay, d	5 [[2], [3], [4], [5], [6], [7]]	7 [[4], [5], [6], [7], [8], [9], [10], [11], [12]]	0.439	<0.001	5 [[2], [3], [4], [5], [6], [7], [8], [9], [10], [11]]	7 [[4], [5], [6], [7], [8], [9], [10], [11], [12]]	0.099	0.01

**Notes:** Data are presented as number (percentage) or median [interquartile range].

**Abbreviations:** AKI, acute kidney injury; AMS, altered mental status; ASD, absolute standardized difference; IUC, indwelling urinary catheter; KDIGO, Kidney Disease Improving Global Outcomes; PACU, post-anesthesia care unit; TIVA, total intravenous anesthesia; UC, urinary catheter; UTI, urinary tract infection.

**Fig. 2:**
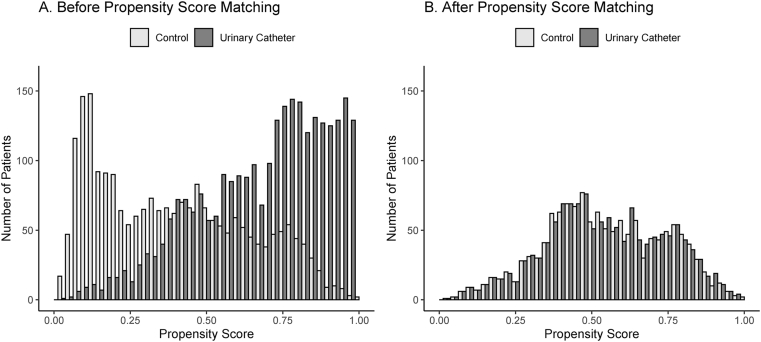
Propensity scores for indwelling urinary catheter (IUC) use in surgical patients. The IUC propensity score is the probability for given baseline variables that any patient in either group would be selected for IUC. (A) Before propensity score matching. (B) After propensity score matching. One-to-one propensity score matching provided 1338 pairs (caliper, 0.1; C-statistic, 0.82 [95% confidence interval, 0.80–0.82]). Abbreviations: IUC, indwelling urinary catheter.

**Fig. 3:**
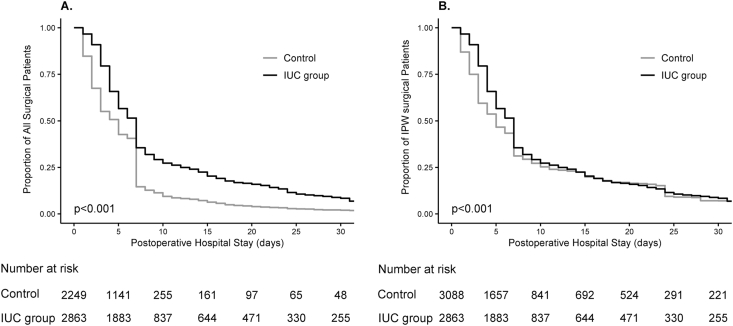
Kaplan-Meier curves for postoperative hospital stay. (A) Kaplan-Meier curves for postoperative hospital stay censored at 30 d after surgery in unmatched surgical patients. The median observation time was 5 d (interquartile range, 2–7 d) for the control group and 7 d (interquartile range, 4–12 d; log-rank test, P < 0.001) for the indwelling urinary catheter (IUC) group. (B) Kaplan-Meier curves for postoperative hospital stay censored at 30 d after surgery in inverse propensity-weighted (IPW) surgical patients. The median observation time was 5 d (interquartile range, 2–11 d) for the control group and 7 d (interquartile range, 4–12 d; log-rank test, P < 0.001) for the IUC group. Abbreviations: IUC, indwelling urinary catheter; IPW, inverse probability weighting.

We present the propensity-scores for IUC in Fig. 2 and the patient characteristics after adjustment with IPW in Table 1 and **2** After weighting, the preoperative characteristics were almost well balanced between the two groups. The actual surgery duration was still longer in the IUC group than in the control group **(ASD = 0.344),** and the prevalence of the primary endpoint was higher in the IUC group **(5.0% vs. 3.2% for the control group, ASD = 0.091; adjusted odds ratio [OR], 1.97; 95% confidence interval [CI] 1.50**–**2.59;** Table 2, Table 3**)**. After IPW, the length of the postoperative hospital stay tended to be longer in the IUC group, and IUC use was again a risk factor of prolonged hospital stay (Table 3; Fig. 3). The incidences of AKI did not differ between the two groups after IPW **(ASD** < **0.001;** Table 2**)**. We performed sensitivity analyses and found that IUC use was associated with the primary endpoint incidence (propensity-matched model, adjusted OR, 1.82; 95% CI, 1.11–2.99; doubly robust-weighted model, adjusted OR, 1.76; 95% CI, 1.34–2.31) and prolonged postoperative hospital stay (propensity-matched model, adjusted hazard ratio, 0.74; 95% CI, 0.68–0.80; doubly robust-weighted model, adjusted hazard ratio, 0.62; 95% CI, 0.59–0.66).

**Table 3:** Multivariable Cox proportional hazards regression model for hospital discharge in unmatched and inverse propensity-weighted patients.

	Hazard ratio (95% CI)			
	All patients	*P*-value	IPW patients	*P*-value
Urinary catheter	0.63 [0.59–0.67]	<0.001	0.84 [0.80–0.89]	<0.001
Age >65 y	0.68 [0.64–0.72]	<0.001	0.63 [0.59–0.66]	<0.001
Male	0.85 [0.80–0.91]	<0.001	0.68 [0.64–0.72]	<0.001
Duration of surgery >120 min	0.64 [0.60–0.68]	<0.001	0.51 [0.48–0.54]	<0.001
Surgery type				
General surgery^a^	Reference		Reference	
ENT surgery^b^	0.46 [0.42–0.50]	<0.001	0.73 [0.68–0.79]	<0.001
Orthopedic surgery^c^	0.28 [0.26–0.30]	<0.001	0.27 [0.25–0.29]	<0.001
Others	0.83 [0.73–0.95]	0.006	1.07 [0.97–1.19]	0.176

Notes.

Abbreviations: CI, confidence interval; ENT, ear, nose, and throat; IPW, inverse propensity-weighted.

a General surgery mainly consisted of cholecystectomy, hernia repair, and mastectomy.

b ENT surgery mainly consisted of septoplasty, tonsillectomy, tympanoplasty, and parotidectomy.

c Orthopedic surgery mainly consisted of treatment for upper or lower limb fracture, arthroplasty or osteotomy of the knee or foot, and spine surgery.

## 4 Discussion

In this retrospective, single-center study, we suggested that intraoperative IUC use is associated with an increased incidence of altered mental status and urinary tract infection.

Our data showed that patients in the IUC group were at a higher risk of postoperative alteration in mental status and less frequently, urinary tract infections. **As we do not assess delirium with diagnostic tools routinely, the possible delirium was described as “altered mental status”.** This made comparisons with previous studies difficult. In our study, the total number of patients with altered mental status was 182 (3.56%). Assuming equivalence of altered mental status and delirium, the altered mental status incidence was lower in this study than in previous studies [20,[28], [29], [30]] because almost all procedures comprised mainly less invasive surgery (Table 1). Agarwal et al. [35] reported that 91% of the patients with IUCs experience discomfort within an hour after surgery; thus, IUC-induced discomfort may have caused altered mental status in our study.

Our study verified the hypothesis that IUC use is associated with altered mental status and urinary tract infection. This finding is supported by other studies. **In a prospective cohort study on 209 patients who underwent noncardiac surgery**, **de Castro *et al.*** [20] **reported that IUC had perioperative risk factors associated** with the development of delirium (incidence ratio, 16.9%; **OR**, 3.7; 95% CI, 1.1–11.4). However, they did not focus on IUC use. Eide et al. [22] verified this hypothesis in 136 patients, but their study population was limited to patients who underwent aortic valve surgery (incidence ratio of delirium, 55.9%; **OR**, 1.04; 95% CI, 1.00–1.08). Our study included various types of surgeries, although they were all minimally invasive procedures. Besides, the population of our study was larger than those of previous studies, thereby validating our findings.

Urinary tract infection is a common IUC complication. A meta-analysis by Saint [31] indicated a 26% pooled cumulative incidence of developing bacteriuria in patients with IUCs for 2–10 d; this bacteriuria incidence accounted to a 3–10% daily estimate. Our study identified two cases of urinary tract infection exclusively in the IUC group (0.06%). We removed the urinary catheters on postoperative days 6 and 2, respectively. Our IUC durations were similar to those in the study by Saint [30], but the urinary tract infection incidence in our study was considerably lower. We speculate that the mean IUC duration might have been shorter in our study as it included less invasive surgeries. However, we were not able to verify this because we could not determine the day of IUC removal.

In our study, hospital stays were longer in the IUC group than in the control group (Table 2, Table 3; Fig. 3). IUCs may lead to delirium or urinary tract infection and restrict the intra- and postoperative management and clinical decision-making process (*e.g.*, completion of the treatment) due to a greater reliance on parameters such as urine output [36]. These may result in prolonged hospital stays.

IUCs are used intraoperatively for urine drainage and to measure urine output. Usually, many anesthesiologists measure urine output to maintain optimal blood pressure [4,[6], [7], [8],[11], [12], [13], [14], [15], [16], [17]]. However, there is little evidence on the management of urine output for improving the prognosis (*e.g.*, decreased mortality) [37,38]. In our study, almost all surgeries were less invasive, had short durations (about 100 min), and had low urine outputs (median volume about 150 mL). This study was inconclusive on whether urine output should be measured for subsequent blood pressure regulation. Further studies regarding blood pressure control based on intraoperative urine output are needed.

Intraoperative oliguria for more than 2 h may predict postoperative AKI [9,10], but this is controversial [9,10,25]. In our study, surgery durations were short and AKI incidences were low. Thus, it was difficult to make clinical judgments on intraoperative oliguria and to predict postoperative AKI. We suggest that IUCs may not be needed to predict postoperative AKI in minimally invasive surgical patients.

Baldini et al. [39] recommend bladder catheterization in outpatients when the bladder volume exceeds 600 mL over 120 min [39]. The CDC suggests that IUCs are appropriate for patients with anticipated intraoperative large-volume infusions or diuretics use [18]. In our study, the median urine output was substantially lower than the bladder capacity. Future studies should assess the association between surgery duration and intraoperative IUC use.

This is the largest observational study focusing on IUCs’ association with complications after minimally invasive surgery. However, this study had some limitations. First, the endpoint “altered mental status” was not based on diagnostic criteria or on a delirium tool such as CAM-ICU. Most previous studies assessing the prevalence of delirium used these screening tools [40]. **The use of variable descriptions for “altered mental state” could have affected the reporting of this occurrence.** Therefore, the descriptions defining “altered mental status” were discussed before the study started and individually assessed to eliminate bias. Second, **most procedures were minimally invasive.** Third, the study included a few patients who underwent major abdominal surgery. We mostly use IUCs in patients with epidural catheters because epidural anesthesia may inhibit parasympathetic neurons of the spinal cord causing urinary retention. We excluded abdominal surgery patients with epidural catheters because IUCs might have been used for the patients. Finally, we did not assess other adverse events such as urethral trauma that might cause severe complications such as urosepsis and AKI [40] because we did not record these urethral traumata systematically.

Our study demonstrated that intraoperative IUC use increases the risk of postoperative altered mental status, urinary tract infection, and prolonged hospital stay. **However, the present results may be confounded by type 1 error because the altered mental status incidence was lower. According to our calculations, a RCT would require a total of 5184 patients (type 1 error** < **0.05 with a power of 0.8). Therefore, we conclude that patients undergoing minimally invasive surgery may not need IUCs during surgery, further studies are needed to evaluate the IUC use preoperatively.**

## Data availability

The data is sharing in a repository in which the authors deposited the data (University Hospital Medical Information Network Individual Case Data Repository [UMIN-ICDR]).

## Ethical approval

The Jikei University School of Medicine Institutional Review Board approved the study (registration number: 31-030 [9529]; principal investigator: Toko Fukushima; date of registration: April 22, 2019).

## Sources of funding

This work was supported by funding from our department. This research did not receive any specific grants from funding agencies in the public, commercial, or not-for-profit sectors.

## Author contribution

1. Toko Fukushima, M.D. Contribution: This author helped design the study, collected and analyzed data, and prepared the manuscript.

2. Kazuhiro Shoji, M.D. Contribution: This author helped design the study, collected data, and prepared the manuscript.

3. Atsuko Tanaka, M.D.

Contribution: This author helped design the study, collected data, and prepared the manuscript.

4. Yukari Aoyagi, M.D.

Contribution: This author helped design the study, collected data, and prepared the manuscript.

5. Seiko Okui, M.D.

Contribution: This author helped design the study, collected data, and prepared the manuscript.

6. Marie Sekiguchi, M.D.

Contribution: This author helped design the study, collected data, and prepared the manuscript.

7. Ayako Shiba, M.D.

Contribution: This author helped design the study, collected data, and prepared the manuscript.

8. Takanori Hiroe M.D., Ph.D. Contribution: This author helped design the study, analyzed the data, and prepared the manuscript.

9. Yasushi Mio, M.D. Contribution: This author prepared the manuscript.

## Registration of research studies

1. Name of the registry: Indwelling catheters increase altered mental status and urinary tract infection risk: a retrospective cohort study.

2. Unique Identifying number or registration ID: UMIN000042664.

(UMIN-CTR, University Hospital Medical Information Network – Clinical Trial Registry, https://upload.umin.ac.jp/cgi-open-bin/ctr/ctr.cgi?function=brows&action=brows&recptno=R000048679&type=summary&language=J).

3. Hyperlink to your specific registration (must be publicly accessible and will be checked):

https://upload.umin.ac.jp/cgi-bin/ctr/ctr_view_reg.cgi?recptno = R000048679.

## Guarantor

Toko Fukushima is the guarantor of this study.

## Consent

We waived the requirement for informed consent because the procedures did not require any additional intervention and because all patient data were anonymous.

## Provenance and peer review

Not commissioned, externally peer-reviewed.

## Declaration of competing interest

The authors disclose no conflicts of interest.
